# Dr. Richard Morris

**Published:** 1908-01

**Authors:** 


Dr. Richard Morris, of Vassar, Mich., died at his home November 27, 1907, of pneumonia, aged 61 years. He graduated at the University of Buffalo in 1870, and served for several years as sheriff, coroner and health officer in his home county and village.

